# Self-Pollinated Types and Ecological Adaptations of the Desert Plant *Gymnocarpos przewalskii*

**DOI:** 10.3390/plants14101410

**Published:** 2025-05-08

**Authors:** Jiaxin Jian, Xueping Chai, Xiaonan Zhao, Zhaoping Yang

**Affiliations:** 1 College of Life Sciences and Technology, Tarim University, Alar 843300, China; jianjx2023@163.com (J.J.); 15228470065@163.com (X.Z.); 2 State Key Laboratory Incubation Base for Conservation and Utilization of Bio-Resource in Tarim Basin, Tarim University, Alar 843300, China; 3 College of Mechanical and Electronic Engineering, Tarim University, Alar 843300, China; chaixp925@163.com

**Keywords:** *Gymnocarpos przewalskii*, flowering progression, autonomous self-pollination, seedling growth, reproductive assurance

## Abstract

In desert plants, outcrossing is frequently limited by pollinator scarcity, leading to a certain percentage of self-fertilization. However, research on the ecological adaptations of self-fertilized seeds remains limited. *Gymnocarpos przewalskii* Maxim, a Tertiary relict plant in the arid deserts of Northwest China, exhibits pronounced self-pollination. In this study, the population of *G. przewalskii* from the fifth regiment of Alar City was selected to explore its self-pollination types, self-pollination percentages, and ecological adaptations. We found that artificially cross-pollinated *G. przewalskii* produced heavier seeds, faster germination, seedlings with greater biomass, and stronger environmental adaptability than self-pollination. However, the frequency of insect visits per flower was less than one. The fruit setting rate of natural pollination was 6.90%, while that of self-pollination was 4.43%, accounting for 64.20% of the natural fruit setting rate. Additionally, *G. pzewalskii*’s filaments elongated rapidly to make the anthers and stigma at the same height before flowering. These characteristics suggest that *G. przewalskii* is capable of autonomous self-pollination and is prior selfing. *Gymnocarpos przewalskii* likely produces a high proportion of the selfing merely to ensure population survival. These findings offer valuable insights into the adaptation of desert plants to the scarcity of pollinators.

## 1. Introduction

Reproduction is one of the most important stages in the life history of plant populations. It is not only the most basic behavioral process of plant reproduction and the continuation of populations but also the key to the renewal of plant populations [1]. Reproduction has long been widely considered by biologists as a central issue of evolution [2,3]. Darwin proposed the reproductive assurance hypothesis, which refers to angiosperms in environments where outcrossing is unlikely to occur due to insufficient pollinators or critically low population densities. Therefore, self-pollination is chosen to safeguard the reproduction of the population, and it is an important factor in the ability of selfing to evolve [4,5,6]. Additionally, hundreds of flowering plants exhibit bet-hedging strategies to ensure long-term survival [7]. For example, mixed mating systems may function as a reproductive bet-hedging strategy [8]. In unpredictable desert environments, where rainfall and temperature fluctuate drastically, plants also employ bet-hedging strategies to ensure long-term survival.

Few plants in nature are absolute self-pollinators; i.e., a certain frequency of outcrossing is often present. Self-pollination includes self-pollination facilitated by pollinators and autonomous self-pollination [9]. Autonomous selfing is prevalent among desert plants. It has been classified into three modes on the basis of the relative timing of the occurrence of self-pollination and cross-pollination [9,10]. Prior selfing refers to self-pollination that occurs before the opportunity for outcrossing arises. Competing selfing occurs when self-pollination and cross-pollination compete simultaneously for the same ovules. Lloyd and Daniel [11] later modified the definition of competing selfing to refer only to the simultaneous occurrence of self-pollination and cross-pollination in the absence of pollinator intervention. Meanwhile, delayed selfing refers to autonomous self-pollination after the loss of outcrossing opportunities and serves as an important reproductive assurance mechanism [12,13,14]. Stephanie Núñez-Hidalgo [15] showed that bat-pollinated bromeliads adapted to pollination limitations by delayed selfing. Many plants, such as *Mimulus nasutus* Greene and *Dendrobium wangliangii* G. W. Hu, C. L. Long & X. H. Jin have evolved adaptations to self-pollination [16,17]. However, no relevant research has been reported on self-pollinated types in the typical desert plant *Gymnocarpos przewalskii*.

*Gymnocarpos przewalskii* Maxim. belongs to the genus *Gymnocarpos* of the family Caryophyllaceae, and is a typical desert plant. In China, this species is distributed across the four major deserts—Taklamakan, Kumutage, Tengger, and Badain Jaran—from west to east, typically thriving in areas where snowmelt or precipitation scours the desert edges. Due to the isolation of these deserts, *G. przewalskii* populations exhibit a patchy distribution [18]. *Gymnocarpos przewalskii* has a simple community structure and a sparse species composition. It is one of the dominant and constructive species in stony desert vegetation [19]. *Gymnocarpos przewalskii* is a relatively rare Tertiary relict plant in the desert region of Central Asia. It is a typical wild plant in the arid desert region of Northwest China, representing xerophytic floral components of the ancient Mediterranean Sea [20,21]. Therefore, *G. przewalskii* holds important scientific value for studying the occurrence, evolution, and climatic changes of deserts in Northwest China and Inner Mongolia, as well as the origins of xerophytic flora components [22]. *Gymnocarpos przewalskii* plays an important role in windbreaks, sand fixation, improving saline-alkaline soils, and maintaining desert ecological balance [23,24]. It developed a series of regulatory mechanisms to withstand adversity stress and showed a strong adaptive capacity in extremely arid and barren desert environments. Qi et al. [25] found that most genes in the *SOD*, *APX*, and *CAT* families showed significant upregulation under NaCl stress. Fu et al. [26] revealed that up to 20% of the *G. przewalskii* genome showed signatures of local adaptation to aridity. In addition, *G. przewalskii* developed adaptive features for arid environments, including thickened epidermal leaf cells, an enhanced cuticle layer, and compactly arranged palisade tissue [27]. However, studies on the reproductive characteristics and progeny adaptation of *G. przewalskii* are relatively few.

Field surveys found that adult *G. przewalskii* plants produced abundant flowers but had a low fruit setting rate. The young twigs and leaves were frequently browsed by cattle, sheep, and camels. Additionally, anthropogenic disturbances further intensified the pressures on sexual reproduction. These factors led to a dramatic decline in the *G. przewalskii* population’s distribution area over recent decades [28,29]. Currently, the population structure and reproductive characteristics of *G. przewalskii* distributed in the Hami Basin, Hexi Corridor, Zhongwei City, and Alxa have been reported [18,30]. Li et al. [30] researched the floral characteristics and breeding system of *G. przewalskii* in Hami, Xinjiang. They found that the breeding system of *G. przewalskii* was facultatively xenogamous and required pollinators. Wang et al. [31] briefly described *G. przewalskii*’s reproductive characteristics. However, an important region for the growth of *G. przewalskii*, Tarim Basin, has been rarely researched.

In this study, we observed the flowering progression, flower morphology, flower-visiting insects, and pollen-to-ovule (P/O) ratio of *G. przewalskii* populations in the fifth regiment of the First Division of Alar, Xinjiang, to understand the self-pollinated types in *G. przewalskii*. In addition, seeds from naturally pollinated, self-pollinated, and cross-pollinated *G. przewalskii* were collected in a bagging experiment. We compared seed characteristics, fruit setting rates, and germination rates under different pollination treatments. The states of seedling growth were observed, and the survival rates were calculated. This study aims to further explore the ecological adaptations of *G. przewalskii* seeds and seedlings. It will also elucidate how these characteristics adapt to desert environments to ensure the reproduction of populations. These findings provide theoretical support for understanding how desert plants adapt their sexual reproductive strategies to harsh environments.

## 2. Results

### 2.1. Floral Traits, Flowering Progression, and P/O Ratio

**Figure 1:**
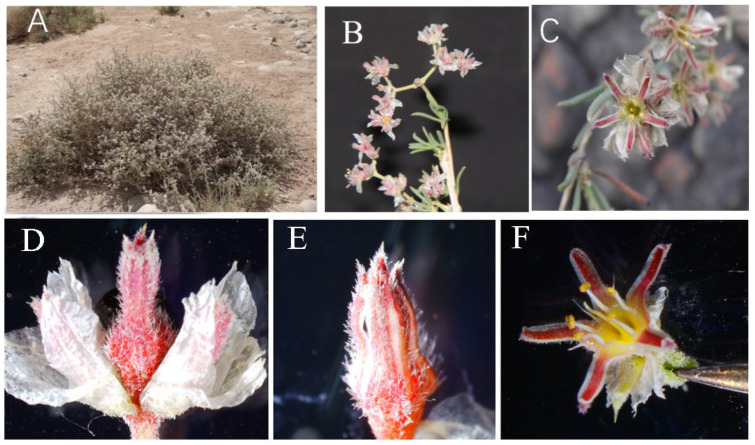
Flowering plant and flowering process of *Gymnocarpos przewalskii*. (**A**) Flowering plant; (**B**) Inflorescence; (**C**) Flower; (**D**) Budding; (**E**) Expanding; (**F**) Flowering.

**Figure 2:**
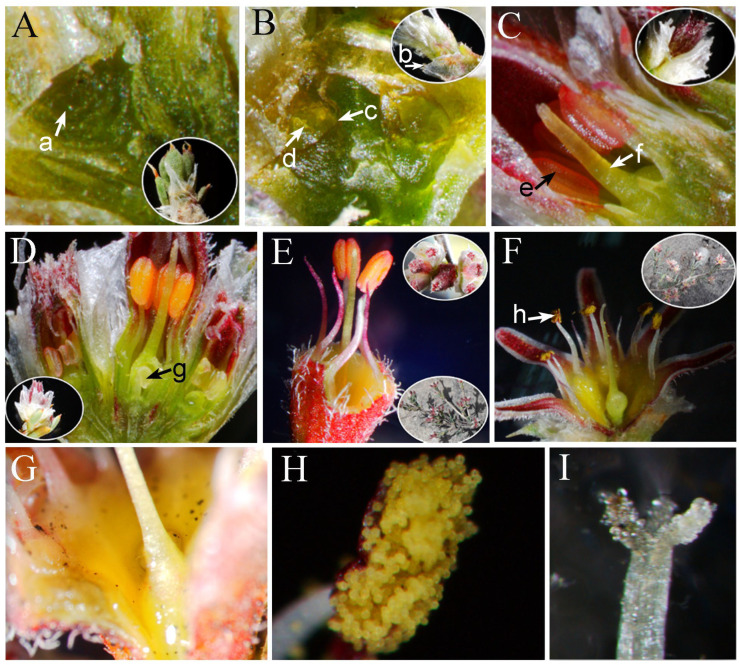
Flower structure in different development stages of *Gymnocarpos przewalskii*. (**A**) 8 April; (**B**) 12 April; (**C**) 19 April; (**D**) 23 April; (**E**) 26 April; (**F**) 30 April; (**G**) Nectary; (**H**) Dehiscent anther; (**I**) Stigma; (a) Flower bud primordium; (b) Bract of the most outer whorl; (c) Calyx tube; (d) Anther; (e) Anther; (f) Style; (g) Oval; (h) Opening anther.

In this study, *G*. *przewalskii* generally began to flower in its third year of growth. The adult plants had a large number of flowers in dichasium inflorescences. The apical flowers bloomed first, followed by sequential opening of the lateral flowers (Figure 1A–C), with a single inflorescence producing up to 31 flowers. In accordance with the flower morphology and developmental process of the stamen and pistil, the blooming process of a single *G. przewalskii* flower could be divided into a budding stage (calyx tube distinctly enlarged, lasting 2–5 d), expanding stage (sepal expansion, lasting 2–4 h) and flowering stage (sepals unfolding, lasting 2–5 d; Figure 1D–F). The developmental period from floral primordium emergence (Figure 2A) to full anthesis spanned approximately one month. The flower developmental order was as follows: bract primordia, sepal primordia, stamen primordia, and pistil primordia (Figure 2B). When the stamens reached a specific developmental stage, the pistil’s stigma elongated rapidly, surpassing the anthers in height (Figure 2C, D). At the expanding stage, the anthers reached the same height as the stigma and slit longitudinally toward the stigma (Figure 2E). The distance between the stigma and anthers was 0.1–3.1 mm, and some pollen often adhered to the stigma (Figure 2F). The receptacle had a certain amount of nectar during the budding stage. The nectar was most abundant during the expanding stage and just after flowering (Figure 2G).

The inflorescences of *G. przewalskii* possessed membranous bracts. The outer whorl bracts were broadly ovate to ovate (Figure 1D), 3.09 ± 0.60 mm in length, and 3.54 ± 0.70 mm in width. The inner bracts were ovate-lanceolate to lanceolate, 3.95 ± 0.45 mm in length, and 2.25 ± 0.52 mm in width. The calyx was dark purplish red, light green, or in between. It was basally united and distally 5-lobed, with corolla degeneration. The flower was 7.69 ± 0.74 mm in diameter. The calyx tube was 1.64 ± 0.26 mm in length, and the calyx lobes were 3.47 ± 0.41 mm in length. The stamens were arranged in two whorls: An outer whorl of five sterile stamens and an inner whorl of five fertile stamens. The anthers of the fertile stamens were dorsifixed. (Figure 2E,F). The filaments measured 2.17 ± 0.19 mm in length, and the anthers were 0.69 ± 0.08 mm long. The pollen was yellow and sub-globose (Figure 2H), with a diameter of 22.74 ± 1.37 μm. The mean pollen production per flower was 9494.8 ± 246.5. The ovary was superior, enclosed within the calyx tube, and measured 4.10 ± 0.22 mm in length. The styles were 3.12 ± 0.16 mm long with 3-lobed stigmas (Figure 2I). The ovule was solitary and inverted (Figure 2D). The P/O ratio was 9494.79 ± 246.51.

### 2.2. Bagging Experiment and Pollinator Observation

**Figure 3:**
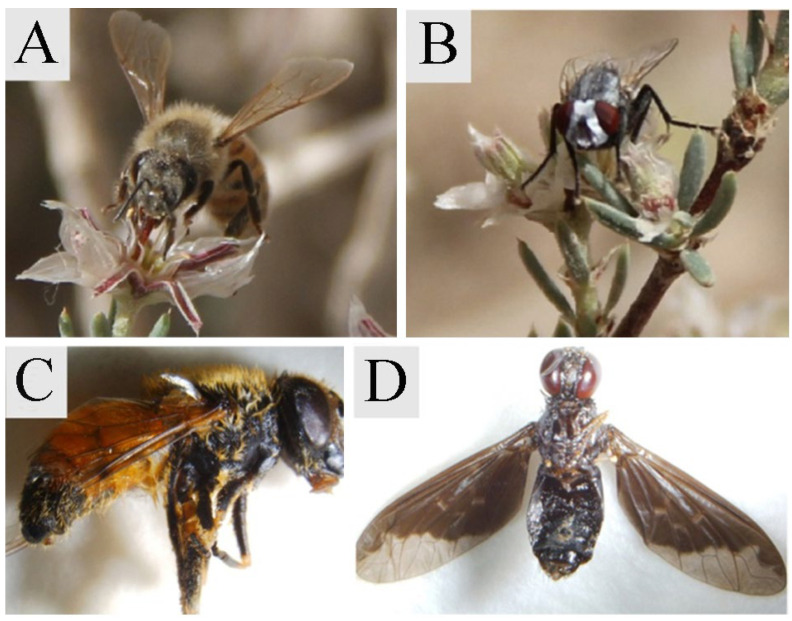
Pollinating insects. (**A**) Apidae; (**B**) Calliphoridae; (**C**) Syrphidae; (**D**) Tabanidae.

Some plastic straws used for bagging could not be collected owing to strong winds in the desert region. The number of fruits and seeds collected is shown in Table 1. The results showed that *G. przewalskii* had no parthenogenesis. The self-pollinated fruit setting rate accounted for 64.20% of the naturally pollinated fruit setting rate. The cross-pollinated fruit setting rate was 30.42%. The field experiments observed four pollinator insects of *G. przewalskii*, which belonged to Apidae, Calliphoridae, Syrphidae, and Tabanidae (Figure 3). The pollinator insects were observed for 5 consecutive days during the flowering stage. We observed that the pollinators visited the flowers during the peak time from 09:00 to 14:00 local time. However, the average visitation frequency was less than one visit per flower.

### 2.3. Biological Characterization of Bagged Fruiting Seeds and Seedlings

#### 2.3.1. Seed Size and Germination Characteristics

Table 2 compares the seed characteristics of *G. przewalskii* under different pollination treatments. The seed length did not differ significantly among artificial cross-pollination, natural pollination, and self-pollination treatments. The seeds from artificial cross-pollination were significantly wider (*p* < 0.05) and heavier (*p* < 0.05) than those from natural pollination and self-pollination. The germination rate of cross-pollination fruiting seeds was higher than that of self-pollination and natural pollination fruiting seeds. The mean germination time of cross-pollination fruiting seeds was shorter than that of self-pollination and natural pollination fruiting seeds.

#### 2.3.2. Seedling Growth

**Figure 4:**
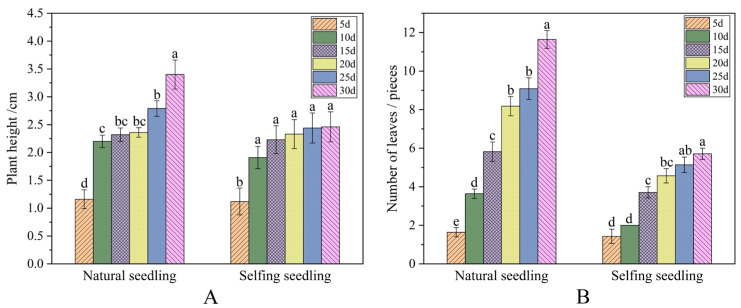
Comparison of the growth state of seedlings from natural pollination and self-pollination seeds of *Gymnocarpos przewalskii*. (**A**) Plant height of natural pollination and self-pollination seedlings; (**B**) The number of leaves of natural pollination and self-pollination seedlings. Note: Different lowercase letters mean significant differences (*p* < 0.05) according to Duncan’s multiple comparison test.

Plant height and leaf number were compared between naturally pollinated and self-pollinated seedlings, as shown in Figure 4. The seedlings from naturally pollinated seeds showed more obvious changes in plant height and leaf number than those from self-pollinated seeds. On Day 30, the plant height of the naturally pollinated seedlings (3.40 ± 0.26 cm) was taller than that of the self-pollinated seedlings (2.46 ± 0.27 cm) (Figure 4A). Meanwhile, the naturally pollinated seedlings had 11–12 leaves (including cotyledons), while the self-pollinated seedlings had only 5–6 leaves (including cotyledons) (Figure 4B).

**Figure 5:**
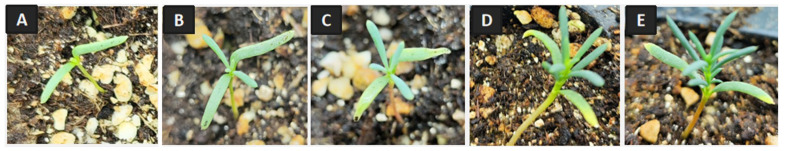
Dynamics of the natural-pollination seed seedling growth process in *Gymnocarpos przewalskii*. (**A**) Day 10; (**B**) Day 15; (**C**) Day 20; (**D**) Day 25; (**E**) Day 30.

**Figure 6:**
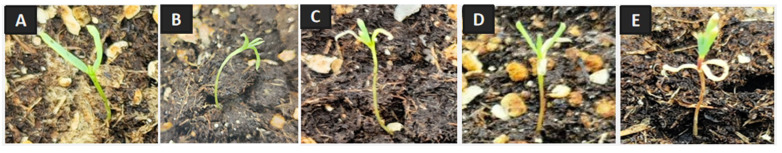
Dynamics of the self-pollination seed seedling growth process in *Gymnocarpos przewalskii*. (**A**) Day 10; (**B**) Day 15; (**C**) Day 20; (**D**) Day 25; (**E**) Day 30.

During the 30 consecutive days of observation, 11 out of the 12 natural pollination seeds germinated (germination rate 91.67%), and all 11 germinated seedlings survived (survival rate 100%). In addition, 8 out of the 12 self-pollination seeds germinated (germination rate 66.67%), and 7 out of the 8 germinated seedlings survived (survival rate 87.5%). The growth process of the seedlings from the naturally pollinated and self-pollinated seeds of *G. przewalskii* is shown in Figure 5 and Figure 6. On Day 30, the cotyledons of the self-pollinated seedlings had withered, and the true leaves gradually turned yellow. By contrast, the natural pollination seedlings had dark green leaves.

## 3. Discussion

### 3.1. Floral Traits and Flowering Progression

Stamens are the male reproductive organs of angiosperms, which are crucial for pollen production and reproductive development. The mature pollen grains contain the plant’s genetic information [32,33]. The pollen and ovule counts are the main factors affecting pollination, limiting the upper and lower limits of seeds. The P/O ratio as a key indicator of the plant breeding system can quickly and easily reflect the breeding system of the phanerogam [34,35,36,37,38,39]. In the present study, the P/O ratio of *G. przewalskii* was 9494.79 ± 246.51. According to Cruden’s [40] P/O ratio criteria, the breeding system of *G. przewalskii* is obligate xenogamy. *Gymnocarpos przewalskii* produces small flowers. It is 7.69 ± 0.74 mm in diameter. The calyx is basally united and distally 5-lobed, with corolla degeneration. Adult plants produce abundant flowers and copious pollen grains, exhibiting the characteristics of both anemophilous and entomophilous plants. Nectar is a floral reward provided by plants to flower visitors. It is an important trait that influences the behavior of flower visitors [41,42]. *Gymnocarpos przewalskii* had nectar glands (Figure 2G), and its nectar is most abundant during the expanding stage and just after flowering. This increases the chances of insect pollination. Nectar volume differs among inflorescence parts, attracting varying numbers of insects and thereby affecting pollination [43]. Zhao et al. [44] reported a gradual decrease in nectar volume from basal to apical flowers in *Aconitum gymnandrum* Maxim., which directed the bumblebee foraging movements upwards. This increased the likelihood of cross-pollination. Whether this is true for *G. przewalskii* still requires extensive experimental proof.

Most desert plants inhabit arid, strongly windy, and cold environments. Under these conditions, habitat fragility and population fragmentation lead to limitations in reproductive pollination and enhanced inbreeding [45,46,47]. *Chenopodium quinoa* Willd. is a predominantly self-pollinating and gynomonoecious species that demonstrates high adaptability to salinity and drought, exhibiting traits such as early flowering and reduced photosynthetic efficiency under stress conditions [48,49]. *Roscoea alpina* Royle and *Roscoea schneideriana* (Loes.) Cowley achieve autonomous self-pollination by bending the style towards the anthers, thus ensuring successful reproduction [50,51]. *Centaurium erythraea* Rafn and *Centaurium littorale* (Turner) Gilmour achieve autonomous self-pollination through anther-curling and dehiscence [52,53]. Several desert plants exhibit self-pollination. For example, *Leontice incerta* Pall. achieves self-pollination by the filaments bending slowly towards the center of the flower after flowering until the corolla (calyx) withers [54]. In *Mimulus verbenaceus* Greene, the senescing epipetalous corolla bends down, abscises, and slides down its style, thereby dragging its anthers across the stigma lobes to ensure self-pollination in desert ecosystems [55]. In the present study, *G. przewalskii* stamens developed before the pistil. When the stamens reached a specific developmental stage, the pistil elongated rapidly to a position where the stigmas were higher than the anthers. Subsequently, rapid filament elongation equalized anther-stigma heights before flowering. The anthers began to release pollens during the expanding stage, and the anthers slit longitudinally towards the stigma. The distance between the stigma and anthers was 0.1–3.1 mm, so the pollens often adhered to the stigma (Figure 2F). *Gymnocarpos przewalskii* achieved autonomous self-pollination, and the type of self-pollination was prior selfing. This mechanism differs from previously reported autonomous self-pollination processes in desert plants. However, the transition from cross-pollination to self-pollination is frequent and diverse in natural systems [56,57,58]. *Gymnocarpos przewalskii* exhibits high adaptive flexibility.

*Gymnocarpos przewalskii* is mainly distributed in the Tarim Basin, Hami Basin, Hexi Corridor, and Edge of the Alxa Desert. It is a relict plant in the desert region of Central Asia [18]. With the uplift of the Qinghai-Tibetan Plateau, the Paratethys Ocean disappeared from Central Asia, and an arid climate began to emerge. During the Quaternary period, climatic shifts enhanced aridification in northwestern regions, driving large-scale desert expansion and consequent habitat fragmentation [22,29]. Since the Late Pleistocene, the extensive development of the Taklamakan Desert has led to the development of desert habitats in the Tarim Basin [59]. *Gymnocarpos przewalskii* has evolved unique genetic adaptations through long-term evolutionary processes to survive the extreme aridity of desert environments. Therefore, *G. przewalskii* transformed its breeding system from cross-pollination to autonomous self-pollination. The rapid elongation of filaments before flowering enables autonomous self-pollination, suggesting a likely reproductive assurance strategy for desert adaptation.

### 3.2. Bagging Experiments and Pollinators

Seeds represent a fundamental evolutionary adaptation in higher plants, serving as the primary vehicle for genetic transmission and ensuring generational reproductive success. As a fundamental requirement for effective plant establishment, they provide the essential basis for population persistence and renewal [60]. However, pollination limitation is a critical constraint to fruit and seed production. For example, *Dendrocalamus sinicus* L. C. Chia & J. L. Sun, *Magnolia stellata* Maxim., and *Penstemon gentianoides* (Kunth) Poir. showed higher fruit setting under artificial cross-pollination [61,62,63]. The present study showed that emasculated *G. przewalskii* flowers that were immediately bagged showed no fruit set (0%), demonstrating the absence of parthenogenesis in this species. The fruit setting rate of natural pollination was 6.90%, while the fruit setting rate of self-pollination was 4.43%, representing 64.20% of the natural fruit setting rate. This result indicates that *G. przewalskii* is capable of self-pollination fruit setting and a high degree of self-adaptation. The fruit setting rate under artificial cross-pollination was 30.42%. This showed that artificial cross-pollination significantly increased the fruit setting rate, revealing substantial pollination limitation. This finding is consistent with previous findings reported by Li et al. [30] for *G. przewalskii* in Hami, Xinjiang.

Field surveys have found four pollinators, belonging to Apidae, Calliphoridae, Syrphidae, and Tabanidae. However, the average visitation frequency was less than one visit per flower, demonstrating that *G. przewalskii* has severe pollinator limitation. Based on the P/O ratio results from this study, *G. przewalskii* exhibits a mixed mating system of self-pollination and cross-pollination, requiring pollinators. This may suggest that *G. przewalskii* employs a bet-hedging strategy to cope with desert environmental stresses. However, *G. przewalskii* showed a low fruit setting rate under both natural and self-pollinated conditions, suggesting constraints from both intrinsic reproductive capacity and extrinsic environmental factors. During the expanding stage, the anthers slit longitudinally in the direction of the stigma, resulting in frequent pollen adhesion on the stigmatic surface. Deposition of inactive pollen on conspecific stigmas may impair female reproductive function through stigma clogging, obstruction of stylar canals, and ovule wastage. Meanwhile, inefficient pollen dispersal to stigmas of the flowers of different plants reduced male reproductive success [64,65]. The impairment of both male and female reproductive functions ultimately caused the reduction in fruit setting. The *G. przewalskii* population of the fifth regiment of Alar City covered an extensive area, comprising approximately 500 plants. However, the community structure was simplified, with sparse species composition. Additionally, the population’s ecological environment exhibited severe fragmentation, and a significant deficiency in pollinators resulted in a markedly low cross-pollination rate. Consequently, *G. przewalskii* adapted to desert environments through self-pollination (fertilization before flowering). However, prolonged reliance on selfing may exacerbate genetic drift within populations, leading to elevated levels of inbreeding depression [66,67].

### 3.3. Seed Germination and Seedling Growth

Inbreeding depression is widespread in plants and has intrigued geneticists and evolutionary biologists since Darwin [68,69,70]. Selfing or inbreeding generates offspring with significantly reduced fitness [71,72,73], manifested through decreased seed quantity and quality. Furthermore, previous studies have demonstrated that self-pollinated offspring show inferior performance compared to cross-pollinated offspring across multiple life stages, including germination, growth, and survival [74,75,76,77,78]. In the present study, the seed characteristics of *G. przewalskii* varied significantly among different pollination treatments. Cross-pollination produced seeds with significantly greater width and single seed weight than natural or self-pollination (Table 2). In the seed germination experiments, cross-pollinated seeds showed a higher germination rate and shorter mean germination time compared to naturally pollinated and self-pollinated seeds. These results indicate that cross-pollinated seeds have higher quality, greater vigor, and better adaptation to arid habitats compared to natural and self-pollinated seeds. To further assess the ecological adaptability of *G. przewalskii* under different pollination treatments, we observed seedling growth performance. The results showed that naturally pollinated seedlings exhibited greater plant height and more leaves than self-pollinated seedlings, indicating their superior growth performance and environmental adaptability. Sexual reproduction, the predominant mode of plant reproduction in nature, primarily involves both self-fertilization (autogamy) and cross-fertilization (allogamy) [79,80]. Therefore, plants produced by cross-pollinated seeds likely have greater environmental adaptability than those from self-pollinated seeds. In the long term, the shift to autonomous self-pollination in *G. przewalskii* may lead to a gradual population decline. Thus, whether *G. przewalskii* can maintain normal reproductive success in the future requires long-term monitoring.

## 4. Materials and Methods

### 4.1. Study Site

This study area was located at the northern edge of the Tarim Basin and the south of the Tianshan Mountains. The region has a warm temperate, highly continental arid (desert) climate. It lies between 40°20′ to 41°47′ N latitude and 79°22′ to 81°53′ E longitude. A population of wild *G. przewalskii* near Gobi Beach, outside Jiamu Town in the fifth regiment of the First Division of Alar, Xinjiang, was selected for this study. The area has minimal human activity and grazing interference from cattle and sheep, reducing potential errors in subsequent bagging experiments.

### 4.2. Floral Traits, Flowering Progression, and P/O Ratio

#### 4.2.1. Observation of Flower Morphology and Flowering Progression

**Figure 7:**
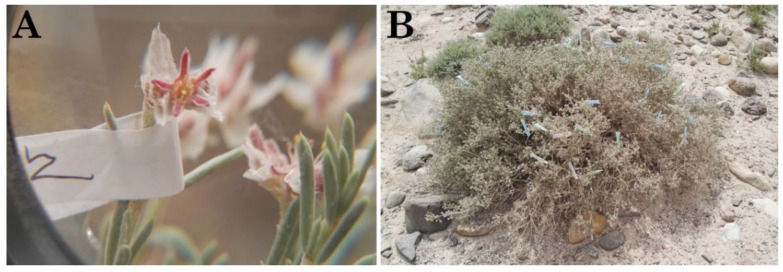
Labeled flower and plant for observing flower characteristics and pollination biology. (**A**) Labeled flower for observing flower characteristics; (**B**) Labeled plant for observing pollination biology.

The wild *G. przewalskii* populations in the fifth regiment of Tarim Basin were observed for two consecutive years (2015 and 2016). The flowering status was recorded every hour during the flowering period. The floral samples of *G. przewalskii* were collected every 3–7 days, from the budding stage to the withering stage, and taken to the laboratory. Then a dissecting microscope was used to observe and measure pollen grain size. Vernier calipers were used to measure floral morphological traits, including inner and outer bracts, calyx dimensions, flower size, ovary length, style length, filament length, and anther length. Each trait was measured in 100 replicates. Additionally, 100 flowers were randomly selected and tagged to record anther dehiscence timing, determining the self-pollination type (Figure 7A).

#### 4.2.2. Determination of P/O Ratio

Pollen quantity per flower was measured using the suspension method [81,82]. Ten *G. przewalskii* plants were randomly selected in the field for this analysis. From each plant, 5 apical flowers were selected. One anther from each flower was excised and immersed in 1% HCl for acid digestion, totaling 5 anthers. The pollen suspension (1 mL) was homogenized by shaker mixing, and 10 replicate 0.01 mL subsamples were extracted using capillary tubes. Pollen grains (n_i_) were counted using a microscope at 10× magnification. The total pollen count per suspension was calculated as 10Σn_i_ (where n_i_ represents the pollen count in each of the 10 aliquots of 0.01 mL). Each *G. przewalskii* flower possesses one ovule and five anthers. The P/O ratio = (pollen per flower)/(ovules per flower). On the basis of Cruden’s [40] criteria, the breeding system was classified into 5 types, with P/O ratios of 2.7–5.4 for cleistogamy, 18.1–39.0 for obligate autogamy, 31.9–396.0 for facultative autogamy, 244.7–2588.0 for facultative xenogamy, and 2108.0–195,525.0 for obligate xenogamy.

### 4.3. Bagging Experiment and Observation of Pollinators

Ten *G*. *przewalskii* plants were randomly selected within the population, with a minimum distance of 10 m apart. For each plant, we randomly selected at least 20 flowers for each direction (east, south, west, and north). A total of at least 80 flowers per plant were bagged for the pollination experiments. To ensure the firmness and ventilation of the bagging wild *G. przewalskii* populations, we used 1-cm-diameter, 5-cm-long transparent plastic tubes (Figure 7B). For ease of use, a 1-cm-long opening was symmetrically cut at one end. Different-colored transparent plastic tubes were used for flowers in each direction of the plants: 5 flowers were emasculated, then bagged, 10 flowers were directly bagged, and 5 flowers were cross-pollinated, then bagged. The pollination treatments were as follows: (1) natural pollination: no bagging, no emasculation, and detection of pollination and fruiting under natural conditions; (2) self-pollination: directly bagging without emasculation to detect the presence of self-incompatibility; (3) no pollination: directly bagging after emasculation to detect whether there was parthenogenesis; and (4) cross-pollination: emasculation and bagging during the budding stage. The bag was opened after one week to conduct pollination on selected flowers. They then were bagged again to detect the fruit setting under cross-pollination. Meanwhile, insect visitation frequency to single flowers was recorded, and pollinator species were observed for 5 consecutive days during the peak activity periods.

### 4.4. Seed Size, Germination Characteristics, and Seedling Growth Experiments

Seeds from natural, self-, and cross-pollinated *G. przewalskii* fruits were collected and brought back to the laboratory. The seeds were manually separated from the sepals. Seed length and width were measured using a stereo microscope (Nikon-SMZ1500, Nikon Corporation, Tokyo, Japan). The single seed weight was determined using an electronic balance (Mettler Toledo ME204E, precision: 0.0001 g, Mettler Toledo, Shanghai, China).

Two filter papers were placed in a 9-cm-diameter Petri dish, and 10 mL of distilled water was added to verify the seed viability of *G. przewalskii* under different pollination treatments. Following Yang et al. [83], the culture conditions were set to 30 °C/35 °C variable temperature and alternating 12 h/12 h of light and darkness. A 5-day germination experiment was conducted on *G. przewalskii*. Water was added on a daily basis to compensate for evaporation. Seed germination was recorded at 12-h intervals. This was based on radicle breaking through the seed coat. Germination percentage (GP) was calculated as: GP = (number of normal germinated seeds at the end of germination/number of tested seeds) × 100% [84]. The mean time of germination was calculated as follows: Σ(Ti × Ni)/ΣNi, where Ni is the number of newly germinated seeds within time Ti [85].

In May 2023, we conducted a supplemented bagging experiment to assess seed ecological adaptation under different pollination treatments in *G. przewalskii*. Seeds from natural and self-pollination were subsequently collected in June. Twelve seeds each from naturally pollinated and self-pollinated *G. przewalskii* were cultivated for thirty days. The plants were grown in a seedling cultivation room at 25 °C under a 12-h light/12-h dark cycle. The seeds were covered with a 0.5 cm soil layer [86], and then with plastic wrap to reduce water evaporation and ensure consistent germination. The growth performance of naturally pollinated and self-pollinated seedlings was monitored every 5 days, with plant survival rate and leaf number recorded at each observation. The seedling survival rate was calculated as (number of surviving seedlings/total number of germinated seeds) × 100% [87].

### 4.5. Data Analysis

Statistical analyses were conducted using SPSS 26.0 (IBM Corp., Armonk, NY, USA). Data were analyzed by one-way ANOVA followed by Duncan’s multiple comparison test (*p* < 0.05). Figures were generated using Origin 2024 (OriginLab Corporation, Northampton, MA, USA).

## 5. Conclusions

*Gymnocarpos przewalskii* flowered before leaf emergence, producing abundant flowers with copious pollen, and rich nectaries that attracted various pollinators, indicating typical cross-pollination characteristics. However, the insect visitation frequency was less than one visit per flower, and bagging experiments showed that autonomous self-pollination accounted for 64.20% of naturally produced seeds in *G. przewalskii*. Therefore, the low fruit setting rate in *G. przewalskii* likely resulted from insufficient pollinator availability within the population. In addition, the flowering process of *G. przewalskii* revealed that its filaments elongated rapidly prior to anther dehiscence, positioning the anthers at the same height as the stigma, indicating a tendency towards autonomous self-pollination. However, germination trials demonstrated superior performance in cross-pollinated progeny, including higher seed mass, faster germination, greater seedling biomass, and improved ecological fitness compared to those from natural and self-pollination. *Gymnocarpos przewalskii* is a xerophytic relic species dating back to the Tertiary period of the ancient Mediterranean flora, now primarily occurring in the desert regions of Central Asia. Its evolutionary adaptation to pollinator scarcity in arid environments likely drove a mating system shift from cross-pollination toward self-pollination. However, seeds from cross-pollination exhibited stronger adaptability. Therefore, we suggested that the production of a greater proportion of autonomously self-pollinated seeds may represent an adaptive reproductive strategy to ensure population persistence under pollinator scarcity in desert environments. Future studies should investigate the reproductive ecology of differently sized *G. przewalskii* populations to determine whether self-pollination serves solely as a reproductive assurance mechanism. Furthermore, to determine whether this reproductive strategy influences *G. przewalskii* population dynamics would require long-term monitoring.

## Figures and Tables

**Table 1:** Fruit setting of *Gymnocarpos przewalskii* under different pollination methods.

Pollination Treatment	Fruit Number	Seed Number	Fruit Setting Rate/%
artificial cross-pollination	192	59	30.42
natural pollination	4000	276	6.90
self-pollination	433	18	4.43
parthenogenesis	188	0	0.00

**Table 2:** Comparison of the seed characteristics of *Gymnocarpos przewalskii* under different pollination treatments.

Pollination Treatment	Seed Length/mm	Seed Width/mm	Single Seed Weight/g	Mean Germination Time/h	Germination Rate/%
artificial cross-pollination	1.77 ± 0.031 a	1.45 ± 0.038 a	2.36 × 10^−3^ ± 0.050 × 10^−3^ a	32.14	94.64
natural pollination	1.75 ± 0.018 a	1.22 ± 0.011 c	1.72 × 10^−3^ ± 0.042 × 10^−3^ b	47.42	92.22
self-pollination	1.75 ± 0.015 a	1.32 ± 0.025 b	1.50 × 10^−3^ ± 0.052 × 10^−3^ c	38.40	83.33

Note: Data are mean ± SD, different lowercase letters mean significant difference at the 5% level.

## Data Availability

All relevant data are included in the manuscript or are available from the corresponding author upon reasonable request.

## References

[1] Wang X. (2024). Invariables and variables in the evolution of plant reproduction. Biol. Divers..

[2] Schoen D.J., Johnson M.T.J., Wright S.I. (2019). The ecology, evolution, and genetics of plant reproductive systems. New Phytol..

[3] Barrett S.C. (2010). Understanding plant reproductive diversity. Philos. Trans. R. Soc. Lond. Ser. B Biol. Sci..

[4] Theologidis I., Chelo I.M., Goy C., Teotónio H. (2014). Reproductive assurance drives transitions to self-fertilization in experimental Caenorhabditis elegans. BMC Biol..

[5] Delgado-Dávila R., Martén-Rodríguez S. (2021). A test of the reproductive assurance hypothesis in Ipomoea hederacea: Does inbreeding depression counteract the benefits of self-pollination?. Am. J. Bot..

[6] Schoen D.J., Morgan M.T., Bataillon T. (1996). How does self-pollination evolve? Inferences from floral ecology and molecular genetic variation. Phil. Trans. R. Soc. Lond. B.

[7] Gianella M., Bradford K.J., Guzzon F. (2021). Ecological, (epi)genetic and physiological aspects of bet-hedging in angiosperms. Plant Reprod..

[8] Falik O., Hoffmann I., Novoplansky A. (2024). A novel type of neighbour perception elicits reproductive plasticity in an annual plant with a mixed mating system. Plant Biol..

[9] Lloyd D.G. (1979). Some Reproductive Factors Affecting the Selection of Self-Fertilization in Plants. Am. Nat..

[10] Brys R., Cauwenberghe J.V., Jacquemyn H. (2016). The importance of autonomous selfing in preventing hybridization in three closely related plant species. J. Ecol..

[11] Lloyd D.G., Schoen D.J. (1992). Self-and cross-fertilization in plants. I. Functional dimensions. Int. J. Plant Sci..

[12] Duan Y.W., Dafni A., Hou Q.Z., He Y.P., Liu J.Q. (2010). Delayed selfing in an alpine biennial *Gentianopsis paludosa* (Gentianaceae) in the Qinghai-Tibetan plateau. J. Integr. Plant Biol..

[13] Goodwillie C., Weber J.J. (2018). The best of both worlds? A review of delayed selfing in flowering plants. Am. J. Bot..

[14] Kalisz S., Vogler D., Fails B., Finer M., Shepard E., Herman T., Gonzales R. (1999). The mechanism of delayed selfing in *Collinsia verna* (Scrophulariaceae). Am. J. Bot..

[15] Núñez-Hidalgo S., Cascante-Marín A. (2024). Selfing in epiphytic bromeliads compensates for the limited pollination services provided by nectarivorous bats in a neotropical montane forest. AoB Plants.

[16] Dole J.A. (1992). REPRODUCTIVE ASSURANCE MECHANISMS IN THREE TAXA OF THE MIMULUS GUTTATUS COMPLEX (SCROPHULARIACEAE). Am. J. Bot..

[17] Wang Q., Shao S., Su Y., Hu X., Shen Y., Zhao D. (2019). A novel case of autogamy and cleistogamy in *Dendrobium wangliangii*: A rare orchid distributed in the dry-hot valley. Ecol. Evol..

[18] Zhang H.-X., Wang Q., Jia S.-W. (2020). Genomic Phylogeography of *Gymnocarpos przewalskii* (Caryophyllaceae): Insights into Habitat Fragmentation in Arid Northwestern China. Diversity.

[19] Li M., He C., Wei M., Long J., Wang J., Yang X., Wang K., He X. (2024). Temporal and spatial dynamics and functional metabolism of dark septate endophytes of *Gymnocarpos przewalskii* Maxim. in Northwest Desert, China. Appl. Soil Ecol..

[20] Li M., He C., Gong F., Zhou X., Wang K., Yang X., He X. (2024). Seasonal and soil compartmental responses of soil microbes of *Gymnocarpos przewalskii* in a hyperarid desert. Appl. Soil Ecol..

[21] Zhao L., Zhang K., Sun X., He X. (2022). Dynamics of arbuscular mycorrhizal fungi and glomalin in the rhizosphere of *Gymnocarpos przewalskii* in Northwest Desert, China. Appl. Soil Ecol..

[22] Jia S.W., Zhang M.L. (2019). Pleistocene climate change and phylogeographic structure of the *Gymnocarpos przewalskii* (Caryophyllaceae) in the northwest China: Evidence from plastid DNA, ITS sequences, and Microsatellite. Ecol. Evol..

[23] Huang H., Cui P., Lu G., Wang X., Jiang L., Luo Y. (2023). Photosynthetic and Antioxidant Responses of *Gymnocarpos przewalskii* to Simulated Rainfall Changes. Forests.

[24] Yang Z., Zhang Y., Pan L., Fu C. (2018). Characterization of the complete chloroplast genome of *Gymnocarpos przewalskii*, an endangered species in China and Mongolia. Conserv. Genet. Resour..

[25] Qi J., Luo Y., Huang H., Lu S., Zhao F., Deng Z., Qiu Y. (2023). Molecular Mechanism of Response and Adaptation of Antioxidant Enzyme System to Salt Stress in Leaves of *Gymnocarpos przewalskii*. Plants.

[26] Fu R., Zhu Y., Liu Y., Yang Z., Lu R., Qiu Y., Lascoux M., Li P., Chen J. (2024). Shared xerophytic genes and their re-use in local adaptation to aridity in the desert plant *Gymnocarpos przewalskii*. Mol. Ecol..

[27] Yang Z., Xu Y., Li Z. (2011). The Leaf blade Anatomical structures and Their Ecological Adaptability of *Gymnocarpos przewalskii* Maxim. J. Anhui Agric. Sci..

[28] Ma S.M., Zhang M.L., Sanderson S.C. (2012). Phylogeography of the rare *Gymnocarpos przewalskii* (Caryophyllaceae): Indications of multiple glacial refugia in north-western China. Aust. J. Bot..

[29] Ma S., Zhang M. (2012). Phylogeography and conservation genetics of the relic *Gymnocarpos przewalskii* (Caryophyllaceae) restricted to northwestern China. Conserv. Genet..

[30] Li X.R., Tang X., Fu W.J. (2016). Floral syndrome and breeding system of *Gymnocarpos przewalskii* Maxim. Chin. J. Ecol..

[31] Wang Z.B., Gao Q.X., Sun J.Z., Ma Q. (2009). Study on Biological Characteristics of Rare Endangered Plant *Gymnocarpos Przewalskii*. Resour. Dev. Mark..

[32] Mao J., Tang Q., Wu H., Chen Y. (2024). Transcriptome Remodeling in *Arabidopsis*: A Response to Heterologous Poplar MSL-lncRNAs Overexpression. Plants.

[33] Shen S., Ma S., Liu Y., Liao S., Li J., Wu L., Kartika D., Mock H.P., Ruan Y.L. (2019). Cell Wall Invertase and Sugar Transporters Are Differentially Activated in Tomato Styles and Ovaries During Pollination and Fertilization. Front. Plant Sci..

[34] Nepal S., Trunschke J., Ren Z.X., Burgess K.S., Wang H. (2023). Community-wide patterns in pollen and ovule production, their ratio (P/O), and other floral traits along an elevation gradient in southwestern China. BMC Plant Biol..

[35] Pías B., Guitián P. (2001). Flowering phenology and pollen-to-ovule ratio in coastal dune communities near Eurosiberian-Mediterranean border in the NW Iberian peninsula. Flora.

[36] Cruden R.W. (2000). Pollen grains: Why so many?. Plant Syst. Evol..

[37] Etcheverry A.V., Alemán M.M., Figueroa-Fleming T., López-Spahr D., Gómez C.A., Yáñez C., Figueroa-Castro D.M., Ortega-Baes P. (2012). Pollen: Ovule ratio and its relationship with other floral traits in *Papilionoideae* (Leguminosae): An evaluation with Argentine species. Plant Biol..

[38] Dobeš C., Milosevic A., Prohaska D., Scheffknecht S., Sharbel T.F., Hülber K. (2013). Reproductive differentiation into sexual and apomictic polyploid cytotypes in *Potentilla puberula* (Potentilleae, Rosaceae). Ann. Bot..

[39] Surina B., Balant M., Glasnović P., Gogala A., Fišer Ž., Satovic Z., Liber Z., Radosavljević I., Classen-Bockhoff R. (2024). Lack of pollinators selects for increased selfing, restricted gene flow and resource allocation in the rare Mediterranean sage Salvia brachyodon. Sci. Rep..

[40] Cruden R.W. (1977). Pollen-Ovule Ratios: A Conservative Indicator of Breeding Systems in Flowering Plants. Evol. Int. J. Org. Evol..

[41] Li D.F., Yan X.C., Lin Y., Wang L., Wang Q. (2021). Do flowers removed of either nectar or pollen attract fewer bumblebee pollinators? An experimental test in *Impatiens oxyanthera*. AoB Plants.

[42] Yamasaki E., Kawakita A., Sakai S. (2013). Modified leaves with disk-shaped nectaries of *Macaranga sinensis* (Euphorbiaceae) provide reward for pollinators. Am. J. Bot..

[43] Chen T., Zhou Y., Zhang J., Peng Y., Yang X., Hao Z., Lu Y., Wu W., Cheng T., Shi J. (2021). Integrative analysis of transcriptome and proteome revealed nectary and nectar traits in the plant-pollinator interaction of Nitraria tangutorum Bobrov. BMC Plant Biol..

[44] Zhao Z., Lu N., Conner J.K. (2016). Adaptive pattern of nectar volume within inflorescences: Bumblebee foraging behavior and pollinator-mediated natural selection. Sci. Rep..

[45] Xu Z., Zhang M.L., Cohen J.I. (2016). Phylogeographic History of Atraphaxis Plants in Arid Northern China and the Origin of A. bracteata in the Loess Plateau. PLoS ONE.

[46] Moody-Weis J.M., Heywood J.S. (2001). Pollination limitation to reproductive success in the Missouri evening primrose, Oenothera macrocarpa (Onagraceae). Am. J. Bot..

[47] Fischer M., Matthies D. (1997). Mating structure and inbreeding and outbreeding depression in the rare plant Gentianella germanica (Gentianaceae). Am. J. Bot..

[48] Zurita-Silva A., Fuentes F., Zamora P., Jacobsen S.E., Schwember A.R. (2014). Breeding quinoa (*Chenopodium quinoa* Willd.): Potential and perspectives. Mol. Breed..

[49] del Pozo A., Ruf K., Alfaro C., Zurita A., Guerra F., Sagredo B. (2023). Traits associated with higher productivity and resilience to drought-prone Mediterranean environments of coastal-lowland quinoa (*Chenopodium quinoa* Willd.). Field Crops Res..

[50] Paudel B.R., Shrestha M., Dyer A.G., Li Q.J. (2017). Ginger and the beetle: Evidence of primitive pollination system in a Himalayan endemic alpine ginger (Roscoea alpina, Zingiberaceae). PLoS ONE.

[51] Zhang Z.Q., Li Q.J. (2008). Autonomous selfing provides reproductive assurance in an alpine ginger *Roscoea schneideriana* (Zingiberaceae). Ann. Bot..

[52] Schouppe D., Brys R., Vallejo-Marin M., Jacquemyn H. (2017). Geographic variation in floral traits and the capacity of autonomous selfing across allopatric and sympatric populations of two closely related Centaurium species. Sci. Rep..

[53] Brys R., Jacquemyn H. (2011). Variation in the functioning of autonomous self-pollination, pollinator services and floral traits in three Centaurium species. Ann. Bot..

[54] Mamut J., Huang D.H., Qiu J., Tan D.Y. (2023). Stamen curvature and temporal flower closure assure reproductive success in an early-spring-flowering perennial in the cold desert of Middle Asia. J. Plant Res..

[55] Vickery R.K. (2008). How Does *Mimulus verbenaceus* (Phrymaceae) Set Seed in the Absence of Pollinators?. Evol. Biol..

[56] Carleial S., van Kleunen M., Stift M. (2017). Relatively weak inbreeding depression in selfing but also in outcrossing populations of North American Arabidopsis lyrata. J. Evol. Biol..

[57] Tsuchimatsu T., Fujii S. (2022). The selfing syndrome and beyond: Diverse evolutionary consequences of mating system transitions in plants. Philos. Trans. R. Soc. London Ser. B Biol. Sci..

[58] Moeller D.A., Geber M.A. (2005). Ecological context of the evolution of self-pollination in Clarkia xantiana: Population size, plant communities, and reproductive assurance. Evol. Int. J. Org. Evol..

[59] Sun J., Zhang L., Deng C., Zhu R. (2008). Evidence for enhanced aridity in the Tarim Basin of China since 5.3 Ma. Quat. Sci. Rev..

[60] Yu S.X., Jiang Y.T., Lin W.H. (2022). Ovule initiation: The essential step controlling offspring number in Arabidopsis. J. Integr. Plant Biol..

[61] Chen L.N., Cui Y.Z., Wong K.M., Li D.Z., Yang H.Q. (2017). Breeding system and pollination of two closely related bamboo species. AoB Plants.

[62] Hirayama K., Ishida K., Tomaru N. (2005). Effects of pollen shortage and self-pollination on seed production of an endangered tree, Magnolia stellata. Ann. Bot..

[63] Salas-Arcos L., Lara C., Ornelas J.F. (2017). Reproductive biology and nectar secretion dynamics of *Penstemon gentianoides* (Plantaginaceae): A perennial herb with a mixed pollination system?. PeerJ.

[64] Barrett S.C. (2002). Sexual interference of the floral kind. Heredity.

[65] Katsuhara K.R., Ushimaru A., Miyazaki Y. (2024). Does a coexisting congener of a mixed mating species affect the genetic structure and selfing rate via reproductive interference?. Oecologia.

[66] Cisternas-Fuentes A., Jogesh T., Broadhead G.T., Raguso R.A., Skogen K.A., Fant J.B. (2022). Evolution of selfing syndrome and its influence on genetic diversity and inbreeding: A range-wide study in Oenothera primiveris. Am. J. Bot..

[67] Cheptou P.O. (2019). Does the evolution of self-fertilization rescue populations or increase the risk of extinction?. Ann. Bot..

[68] Han T., Wang F., Song Q., Ye W., Liu T., Wang L., Chen Z.J. (2021). An epigenetic basis of inbreeding depression in maize. Sci. Adv..

[69] Porcher E., Lande R. (2016). Inbreeding depression under mixed outcrossing, self-fertilization and sib-mating. BMC Evol. Biol..

[70] Ahlinder J., Giles B.E., García-Gil M.R. (2021). Life stage-specific inbreeding depression in long-lived Pinaceae species depends on population connectivity. Sci. Rep..

[71] Brandvain Y., Thomson L., Pyhäjärvi T. (2024). Early-acting inbreeding depression can evolve as an inbreeding avoidance mechanism. Proc. Biol. Sci..

[72] Cheptou P.O. (2024). The evolutionary ecology of inbreeding depression in wild plant populations and its impact on plant mating systems. Front. Plant Sci..

[73] Wright S.I., Kalisz S., Slotte T. (2013). Evolutionary consequences of self-fertilization in plants. Proc. Biol. Sci..

[74] Lu J., Yi H., Tan D., Baskin C.C., Baskin J.M. (2022). Germination of Seeds from Flowers along a Continuum of Long to Short Styles in the Cold Desert Perennial Herb *Ixiolirion songaricum*. Plants.

[75] Xu Y.W., Sun L., Ma R., Gao Y.Q., Sun H., Song B. (2023). Does pollinator dependence decrease along elevational gradients?. Plant Divers..

[76] Sheridan M.P., Karowe N.D. (2000). Inbreeding, outbreeding, and heterosis in the yellow pitcher plant, Sarracenia flava (Sarraceniaceae), in Virginia. Am. J. Bot..

[77] Donohue K. (1998). Effects of inbreeding on traits that influence dispersal and progeny density in Cakile edentula var. lacustris (Brassicaceae). Am. J. Bot..

[78] Snell R., Aarssen L.W. (2005). Life history traits in selfing versus outcrossing annuals: Exploring the ‘time-limitation’ hypothesis for the fitness benefit of self-pollination. BMC Ecol..

[79] Hanschen E.R., Herron M.D., Wiens J.J., Nozaki H., Michod R.E. (2018). Repeated evolution and reversibility of self-fertilization in the volvocine green algae. Evol. Int. J. Org. Evol..

[80] Bressan E.D., Sebbenn A.M., Ferreira R.R., Lee T.S., Figueira A. (2013). *Jatropha curcas* L. (Euphorbiaceae) exhibits a mixed mating system, high correlated mating and apomixis. Tree Genet. Genomes.

[81] Wang Y., Meng L.L., Yang Y.P., Duan Y.W. (2010). Change in floral orientation in *Anisodus luridus* (Solanaceae) protects pollen grains and facilitates development of fertilized ovules. Am. J. Bot..

[82] He J., Dong T., Huang K., Yang Y., Li D., Xu X., He X. (2017). Sex-specific floral morphology, biomass, and phytohormones associated with altitude in dioecious *Populus cathayana* populations. Ecol. Evol..

[83] Yang Z.P., Liang J.Y., Chai X.P., Xue W.T. (2017). Influence of Environmental Factors on Seed Germination of Endangered Plant *Gymnocarpos przewalskii*. J. Southwest For. Univ. Nat. Sci..

[84] Cheng X., Yao H., Cheng Z., Tian B., Gao C., Gao W., Yan S., Cao J., Pan X., Lu J. (2022). The Wheat Gene TaVQ14 Confers Salt and Drought Tolerance in Transgenic *Arabidopsis thaliana* Plants. Front. Plant Sci..

[85] Vandelook F., Newton R.J., Bobon N., Bohley K., Kadereit G. (2021). Evolution and ecology of seed internal morphology in relation to germination characteristics in Amaranthaceae. Ann. Bot..

[86] Zhang Q., Zhang L., Wang L.X. (2020). Response of seed germination to sowing depth in *Gymnocarpos przewalskii*. Rural. Econ. Sci. Technol..

[87] Guo Z., Wang H., Yao J., Cheng Y., Zhang W., Xu Z., Li M., Huang J., Zhao M. (2022). Quantitative Trait Loci Mapping Analysis for Cold Tolerance Under Cold Stress and Brassinosteroid-Combined Cold Treatment at Germination and Bud Burst Stages in Rice. Front. Plant Sci..

